# Accelerating Progress Towards the 2030 Neglected Tropical Diseases Targets: How Can Quantitative Modeling Support Programmatic Decisions?

**DOI:** 10.1093/cid/ciae082

**Published:** 2024-04-25

**Authors:** Andreia Vasconcelos, Jonathan D King, Cláudio Nunes-Alves, Roy Anderson, Daniel Argaw, Maria-Gloria Basáñez, Shakir Bilal, David J Blok, Seth Blumberg, Anna Borlase, Oliver J Brady, Raiha Browning, Nakul Chitnis, Luc E Coffeng, Emily H Crowley, Zulma M Cucunubá, Derek A T Cummings, Christopher Neil Davis, Emma Louise Davis, Matthew Dixon, Andrew Dobson, Louise Dyson, Michael French, Claudio Fronterre, Emanuele Giorgi, Ching-I Huang, Saurabh Jain, Ananthu James, Sung Hye Kim, Klodeta Kura, Ana Lucianez, Michael Marks, Pamela Sabina Mbabazi, Graham F Medley, Edwin Michael, Antonio Montresor, Nyamai Mutono, Thumbi S Mwangi, Kat S Rock, Martha-Idalí Saboyá-Díaz, Misaki Sasanami, Markus Schwehm, Simon E F Spencer, Ariktha Srivathsan, Robert S Stawski, Wilma A Stolk, Samuel A Sutherland, Louis-Albert Tchuem Tchuenté, Sake J de Vlas, Martin Walker, Simon J Brooker, T Déirdre Hollingsworth, Anthony W Solomon, Ibrahima Socé Fall

**Affiliations:** Big Data Institute, Li Ka Shing Centre for Health Information and Discovery, Nuffield Department of Medicine, University of Oxford, Old Road Campus, Oxford, United Kingdom; Centre for Global Health Research, Nuffield Department of Medicine, University of Oxford, Old Road Campus, Oxford, United Kingdom; Global Neglected Tropical Diseases Programme, World Health Organization, Geneva, Switzerland; Big Data Institute, Li Ka Shing Centre for Health Information and Discovery, Nuffield Department of Medicine, University of Oxford, Old Road Campus, Oxford, United Kingdom; London Centre for Neglected Tropical Disease Research, London, United Kingdom; Department of Infectious Disease Epidemiology, School of Public Health, Faculty of Medicine, St Mary's Campus, Imperial College London, London, United Kingdom; MRC Centre for Global Infectious Disease Analysis, School of Public Health, Imperial College London, London, United Kingdom; Global Neglected Tropical Diseases Programme, World Health Organization, Geneva, Switzerland; London Centre for Neglected Tropical Disease Research, London, United Kingdom; Department of Infectious Disease Epidemiology, School of Public Health, Faculty of Medicine, St Mary's Campus, Imperial College London, London, United Kingdom; MRC Centre for Global Infectious Disease Analysis, School of Public Health, Imperial College London, London, United Kingdom; Department of Biological Sciences, University of Notre Dame, Notre Dame, Indiana, USA; Department of Public Health, Erasmus MC, University Medical Center Rotterdam, Rotterdam, The Netherlands; Francis I. Proctor Foundation, University of California, San Francisco, California, USA; Department of Biology, University of Oxford, Oxford, United Kingdom; Department of Infectious Disease Epidemiology, Faculty of Epidemiology and Population Health, London School of Hygiene & Tropical Medicine, London, United Kingdom; Centre for the Mathematical Modelling of Infectious Diseases, London School of Hygiene & Tropical Medicine, London, United Kingdom; The Department of Statistics, The University of Warwick, Coventry, United Kingdom; Department of Epidemiology and Public Health, Swiss Tropical and Public Health Institute, Allschwil, Switzerland; University of Basel, Basel, Switzerland; Department of Public Health, Erasmus MC, University Medical Center Rotterdam, Rotterdam, The Netherlands; Zeeman Institute for System Biology and Infectious Disease Epidemiology Research, The University of Warwick, Coventry, United Kingdom; Mathematics Institute, The University of Warwick, Coventry, United Kingdom; Departamento de Epidemiología Clínica y Bioestadística, Facultad de Medicina, Universidad Pontificia Javeriana, Bogotá, Colombia; Department of Biology, University of Florida, Gainesville, Florida, USA; Emerging Pathogens Institute, University of Florida, Gainesville, Florida, USA; Zeeman Institute for System Biology and Infectious Disease Epidemiology Research, The University of Warwick, Coventry, United Kingdom; Mathematics Institute, The University of Warwick, Coventry, United Kingdom; Mathematics Institute, The University of Warwick, Coventry, United Kingdom; London Centre for Neglected Tropical Disease Research, London, United Kingdom; Department of Infectious Disease Epidemiology, School of Public Health, Faculty of Medicine, St Mary's Campus, Imperial College London, London, United Kingdom; MRC Centre for Global Infectious Disease Analysis, School of Public Health, Imperial College London, London, United Kingdom; Department of Ecology and Evolutionary Biology, Princeton University, Princeton, New Jersey, USA; Zeeman Institute for System Biology and Infectious Disease Epidemiology Research, The University of Warwick, Coventry, United Kingdom; Mathematics Institute, The University of Warwick, Coventry, United Kingdom; Schistosomiasis Control Initiative, Department of Infectious Disease Epidemiology, St Mary's Campus, Imperial College London, London, United Kingdom; RTI International, Washington, D.C., USA; CHICAS, Lancaster Medical School, Lancaster University, Lancaster, United Kingdom; CHICAS, Lancaster Medical School, Lancaster University, Lancaster, United Kingdom; Zeeman Institute for System Biology and Infectious Disease Epidemiology Research, The University of Warwick, Coventry, United Kingdom; Mathematics Institute, The University of Warwick, Coventry, United Kingdom; Global Neglected Tropical Diseases Programme, World Health Organization, Geneva, Switzerland; Department of Public Health, Erasmus MC, University Medical Center Rotterdam, Rotterdam, The Netherlands; Global Neglected Tropical Diseases Programme, World Health Organization, Geneva, Switzerland; London Centre for Neglected Tropical Disease Research, London, United Kingdom; Department of Infectious Disease Epidemiology, School of Public Health, Faculty of Medicine, St Mary's Campus, Imperial College London, London, United Kingdom; MRC Centre for Global Infectious Disease Analysis, School of Public Health, Imperial College London, London, United Kingdom; Communicable Diseases Prevention, Control, and Elimination, Pan American Health Organization, Washington D.C., USA; Faculty of Infectious and Tropical Diseases, London School of Hygiene & Tropical Medicine, London, United Kingdom; Global Neglected Tropical Diseases Programme, World Health Organization, Geneva, Switzerland; Department of Infectious Disease Epidemiology, Faculty of Epidemiology and Population Health, London School of Hygiene & Tropical Medicine, London, United Kingdom; College of Public Health, University of South Florida, Tampa, Florida, USA; Global Neglected Tropical Diseases Programme, World Health Organization, Geneva, Switzerland; Centre for Epidemiological Modelling and Analysis, University of Nairobi, Nairobi, Kenya; Paul G. Allen School for Global Health, Washington State University, Pullman, Washington, USA; Centre for Epidemiological Modelling and Analysis, University of Nairobi, Nairobi, Kenya; Paul G. Allen School for Global Health, Washington State University, Pullman, Washington, USA; Institute of Immunology and Infection Research, University of Edinburgh, Edinburgh, United Kingdom; Zeeman Institute for System Biology and Infectious Disease Epidemiology Research, The University of Warwick, Coventry, United Kingdom; Mathematics Institute, The University of Warwick, Coventry, United Kingdom; Communicable Diseases Prevention, Control, and Elimination, Pan American Health Organization, Washington D.C., USA; Lancaster Medical School, Lancaster University, Lancaster, United Kingdom; ExploSYS GmbH, Interdisciplinary Institute for Exploratory Systems, Leinfelden-Echterdingen, Germany; Centre for the Mathematical Modelling of Infectious Diseases, London School of Hygiene & Tropical Medicine, London, United Kingdom; Francis I. Proctor Foundation, University of California, San Francisco, California, USA; Institute of Public Health and Wellbeing, School of Health and Social Care, University of Essex, Essex, United Kingdom; Department of Public Health, Erasmus MC, University Medical Center Rotterdam, Rotterdam, The Netherlands; Zeeman Institute for System Biology and Infectious Disease Epidemiology Research, The University of Warwick, Coventry, United Kingdom; Warwick Medical School, The University of Warwick, Coventry, United Kingdom; Centre for Schistosomiasis and Parasitology, Faculty of Sciences, University of Yaounde, Yaounde, Cameroon; Department of Public Health, Erasmus MC, University Medical Center Rotterdam, Rotterdam, The Netherlands; London Centre for Neglected Tropical Disease Research, London, United Kingdom; Department of Pathobiology and Population Sciences, Royal Veterinary College, University of London, London, United Kingdom; Bill & Melinda Gates Foundation, Seattle, Washington, USA; Big Data Institute, Li Ka Shing Centre for Health Information and Discovery, Nuffield Department of Medicine, University of Oxford, Old Road Campus, Oxford, United Kingdom; Global Neglected Tropical Diseases Programme, World Health Organization, Geneva, Switzerland; Global Neglected Tropical Diseases Programme, World Health Organization, Geneva, Switzerland

**Keywords:** neglected tropical diseases, mathematical models, elimination, control, policy-making

## Abstract

Over the past decade, considerable progress has been made in the control, elimination, and eradication of neglected tropical diseases (NTDs). Despite these advances, most NTD programs have recently experienced important setbacks; for example, NTD interventions were some of the most frequently and severely impacted by service disruptions due to the coronavirus disease 2019 (COVID-19) pandemic. Mathematical modeling can help inform selection of interventions to meet the targets set out in the NTD road map 2021–2030, and such studies should prioritize questions that are relevant for decision-makers, especially those designing, implementing, and evaluating national and subnational programs. In September 2022, the World Health Organization hosted a stakeholder meeting to identify such priority modeling questions across a range of NTDs and to consider how modeling could inform local decision making. Here, we summarize the outputs of the meeting, highlight common themes in the questions being asked, and discuss how quantitative modeling can support programmatic decisions that may accelerate progress towards the 2030 targets.

## Teaser

A stakeholder meeting hosted by the World Health Organization served to identify priority modeling questions across a range of neglected tropical diseases (NTDs) that can support programmatic decisions to accelerate progress towards the 2030 targets.

Neglected tropical diseases (NTDs) are a group of conditions with diverse etiologies, including viral, bacterial, fungal, and parasitic infections, which are responsible for substantial health, economic, and social costs in more than 2 billion people [1] (Table 1). While NTDs are varied and their epidemiology is often complex, these diseases primarily afflict low-income communities in tropical or subtropical areas, where they are linked to a range of long-term morbidities and irreversible disabilities that perpetuate the cycle of poverty [2].

**Table 1.** Brief Summary of the Neglected Tropical Diseases Currently Prioritized by the World Health Organization

NTD	Etiology	Control Strategies	2030 Target
Buruli ulcer	Bacterial	IDM	Control
Chagas disease	Parasitic: protozoan	IDM/VC/WASH	EPHP
Dengue	Viral	VC/WASH	Control
Dracunculiasis (Guinea-worm disease)	Parasitic: helminth	VC/VPH/WASH	Eradication
Echinococcosis	Parasitic: helminth	VPH/WASH	Control
Foodborne trematodiases	Parasitic: helminth	PCT/VC/VPH/WASH	Control
Human African trypanosomiasis (sleeping sickness)	Parasitic: protozoan	IDM/VC/VPH/WASH	*Rhodesiense*: EPHP *Gambiense*: EOT
Leishmaniasis	Parasitic: protozoan	IDM/VC/VPH	Cutaneous: control Visceral: EPHP
Leprosy (Hansen's disease)	Bacterial	PCT/WASH	EOT
Lymphatic filariasis	Parasitic: helminth	PCT/VC/WASH	EPHP
Mycetoma, chromoblastomycosis, and other deep mycoses	Fungal/bacterial	Long-term treatment with antibiotics/antifungals, WASH	Control
Onchocerciasis (river blindness)	Parasitic: helminth	PCT/VC	EOT
Rabies	Viral	VPH/WASH	EPHP
Scabies and other ectoparasitoses	Parasitic: arthropod	PCT/WASH	Control
Schistosomiasis	Parasitic: helminth	PCT/VC/VPH/WASH	EPHP
Soil-transmitted helminthiases	Parasitic: helminth	PCT/WASH	EPHP
Snakebite envenoming	Noncommunicable	IDM	Control
Taeniasis/cysticercosis	Parasitic: helminth	PCT/VPH/WASH	Control
Trachoma	Bacterial	PCT/VC/WASH	EPHP
Yaws	Bacterial	IDM/PCT/WASH	Eradication

WHO recommends 5 core strategic interventions to accelerate the prevention, control, elimination, and eradication of NTDs: innovative and intensified disease management (IDM), preventive chemotherapy (PCT), vector (or snail) control (VC), veterinary public health (VPH), and provision of safe water, sanitation, and hygiene (WASH). The 2030 targets are defined by WHO as follows [2]: Control: “Reduction of disease incidence, prevalence, morbidity and/or mortality to a locally acceptable level as a result of deliberate efforts; continued interventions are required to maintain the reduction. Control may or may not be related to global targets set by WHO.” Elimination (interruption of transmission [EOT]): “Reduction to zero of the incidence of infection caused by a specific pathogen in a defined geographical area, with minimal risk of reintroduction, as a result of deliberate efforts; continued action to prevent re-establishment of transmission may be required. Documentation of elimination of transmission is called verification.” Elimination as a public health problem (EPHP): “A term related to both infection and disease, defined by achievement of measurable targets set by WHO in relation to a specific disease. When reached, continued action is required to maintain the targets and/or to advance interruption of transmission. Documentation of elimination as a public health problem is called validation.”

Abbreviations: NTD, neglected tropical disease; WHO, World Health Organization.

In 2012, the World Health Organization (WHO) published its first NTD roadmap 2012–2020 [3], an ambitious plan for the control, elimination, and eradication of 17 NTDs by 2020. This roadmap inspired the London Declaration on NTDs [4], in which a variety of stakeholders pledged support via the donation of medicines, other health products, and funding. Substantial progress followed over the subsequent decade, including the elimination of at least 1 NTD in 42 countries, 600 million fewer people requiring interventions against NTDs than in 2010, and considerable reductions in NTD-related morbidity [1, 5–7]. However, most 2020 targets were not met, prompting WHO to convene interested parties to produce a second NTD road map [2], which was endorsed by the 73rd World Health Assembly in November 2020. Commonly known as the NTD road map for 2021–2030, it provides revised cross-cutting and disease-specific targets for 2030 and identifies critical gaps and actions required to reach those targets. Importantly, the 2030 road map not only aligns NTD targets with the United Nations’ Sustainable Development Goals but also establishes 3 pillars to support the control, elimination, and eradication of NTDs: (1) accelerating programmatic action, (2) intensifying cross-cutting approaches, and (3) changing operating models and culture to facilitate country ownership [2].

By design, the 2021–2030 road map was developed with input from a range of stakeholders, including disease experts and modelers. Infectious disease modelling is playing an increasing role as a tool for understanding, projecting, and forecasting the dynamics of NTDs under different interventions. Furthermore, models can inform the design of optimally effective intervention and surveillance strategies and the testing and roll-out of new tools [7]. WHO specifically engaged the NTD modeling community during the open consultation on the development of the 2021–2030 road map to gain insight into the achievability, measurability, and feasibility of the 2030 targets with current tools, and the identification of risks to be mitigated [8]. More recently, WHO and the NTD Modelling Consortium (https://www.ntdmodelling.org/ ) collaborated to project the impact of disruptions due to the coronavirus disease 2019 (COVID-19) pandemic on NTD programs and to estimate the impact of remedial strategies that could help such programs get back on track [9–18].

While modeling can undoubtedly help to address many questions related to NTD dynamics, interventions, and surveillance, there is a growing appreciation of the need for models to provide high-quality information that can effectively support on-the-ground decision making across NTD programs. National health ministries face diverse challenges, including reduced domestic revenues, locally increased transmission rates due to COVID-19–related interruptions, cuts to NTD implementation funding from donors, and the challenges related to climate change, all of which threaten to slow down progress towards public health goals. Furthermore, given the road map's emphasis on promoting country ownership of programs, there is an increasing need not just to incorporate national and subnational contexts into models but also to strengthen capacity for modeling in institutions within NTD-endemic countries, which are likely to increase the local relevance of model outputs.

To discuss these issues, WHO and the NTD Modelling Consortium organized a meeting in September 2022 at which priority modeling questions for NTDs were identified. Here, we summarize the outputs of the meeting, highlighting pressing questions that could be addressed in whole or in part through modeling, and discuss how models may best support programmatic decisions to meet the 2030 targets.

## MEETING

The meeting took place on 21–22 September 2022 and involved representatives of WHO, the NTD Modelling Consortium, funders, and other stakeholders. Plenary sessions were interspersed with breakout group discussions dedicated to identifying priority modeling questions for specific diseases. A list of participants, the meeting's agenda, and a table with the main questions identified for each session are included in the Supplementary Materials 1–3. The most pressing issues are summarized below.

### Modeling the Impact of the COVID-19 Pandemic

One key area identified as being very likely to benefit from modeling insights was the impact of disruptions to NTD programs due to COVID-19. NTD interventions were some of the health services most severely affected by the pandemic, due to both direct impacts on service delivery as well as indirect impacts including resource re-allocation [18, 19]. While WHO and the NTD Modelling Consortium have previously collaborated to determine how COVID-19–related disruptions impacted NTD programs and to estimate how different strategies could assist in program recovery [9, 18], the meeting highlighted several additional areas that require further studies. For example, participants discussed how modeling may help estimate when visceral leishmaniasis (VL) outbreaks could occur due to COVID-19–related program interruptions, and to assess how trachoma elimination has been affected by the pandemic's impact on availability of medicines, which caused missed rounds and reduced coverage of mass drug administration (MDA) campaigns. Importantly, models need to be tailored to local conditions, incorporating available information about the number of missed MDA rounds and declines in coverage. Furthermore, NTD program managers pointed out that, although routine data reveal a recent decrease in VL reported cases, it is unclear whether these numbers reflect curtailed transmission, reduced availability of clinical services, or lower rates of presenting to healthcare services. While the pandemic resulted in service disruption, not all COVID-19 impacts have been negative as other aspects (such as movement restrictions) can slow transmission, as shown for dengue [20]. Therefore, it will be important to incorporate these factors into existing models to improve their accuracy. Similarly, for trachoma, it was discussed how published models are mainly based on generalized scenarios that focus on the impact of COVID-19 on missed MDA rounds. Rounds were not always completely missed, but coverage was lower after the onset of the pandemic. In such cases, revised models could help not only to better estimate the impact of disruptions to interventions but also help with decision making, such as whether planned impact surveys should go ahead as scheduled. The cost of doing surveys was noted [21], with program managers pointing out that guidance on estimating how likely a district is to pass an impact survey [22], given COVID-19–related challenges, would be invaluable. Finally, it was also noted that the lessons learned from modeling COVID-19–related disruptions will likely be useful for future analyses, including to better understand how future service interruptions that divert essential financial and human resources (due, eg, to outbreaks or threatened outbreaks of disease) may impact NTD programs.

### Incorporating Real-Life Challenges

Modeling may also help to adjust current NTD intervention regimens, particularly by incorporating challenges that are encountered by national and subnational programs, such as decisions on when to start/stop interventions, the impact of never-treated populations, and the cost of surveys and of implementing alternative treatment strategies to accelerate progress towards agreed targets.

#### Start and Stop Decisions

The potential of modeling studies to inform decisions about optimal timings to start and/or stop interventions was discussed in the context of multiple NTDs. For example, for foodborne trematode (FBT) infections, modeling could be used to determine appropriate prevalence and intensity thresholds for recommending the start of MDA campaigns. Similarly, for soil-transmitted helminthiases (STH), revised models could help to inform when and how treatment should be expanded from the 3 priority groups (preschool-aged children, school-aged children, and women of childbearing age) to also include adult populations. The STH models could also be extended to estimate the number and optimal frequency of MDA rounds needed to achieve 2030 targets, and how these vary by transmission setting. Serological thresholds for starting and stopping MDA for onchocerciasis would also benefit from additional modeling, including seroprevalence measured by different tests and in different age groups.

**Figure 1.**
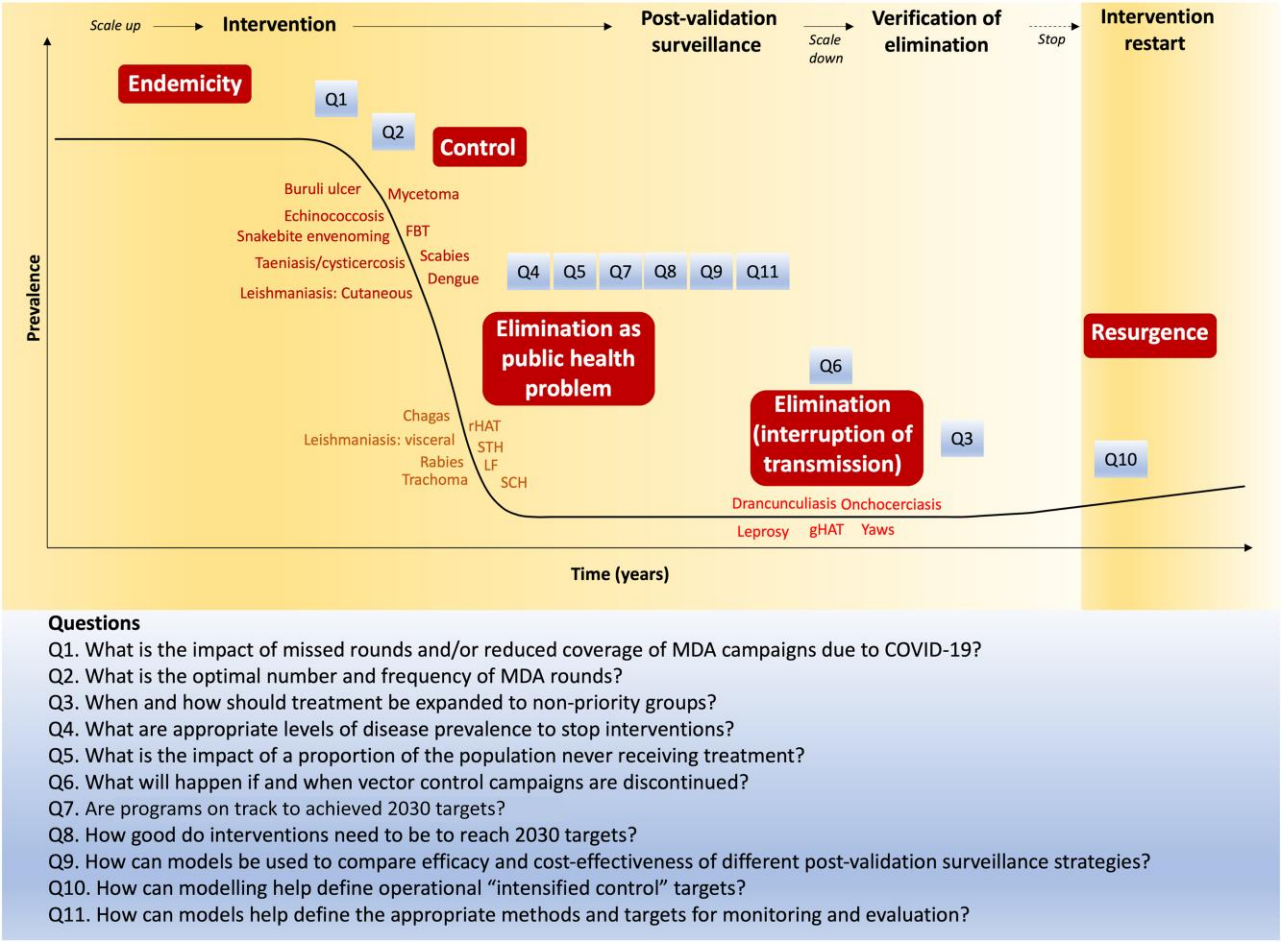
Schematic with the different stages from control to elimination, mapping where different NTDs sit on this spectrum, and where the priority questions from Table 2 sit across these different stages. Abbreviations: COVID-19, coronavirus disease 2019; FBT, foodborne trematode; gHAT, *gambiense* human African trypanosomiasis; LF, lymphatic filariasis; MDA, mass drug administration; NTD, neglected tropical disease; Q, question; rHAT, *rhodes*iense** human African trypanosomiasis; STH, soil-transmitted helminthiases.

The potential utility of modeling to guide decisions around when to stop interventions was also discussed in the context of various diseases given the different types of 2030 targets for some NTDs—for example, elimination as a public health problem (EPHP) or elimination of transmission (EOT), which require different levels of certainty (Table 1, Figure 1). A common thread was the need for models to appropriately quantify the risk of resurgence after stopping treatment and how this should be balanced with the high costs of ongoing treatment, which are not sustainable in resource-constrained settings. For example, for onchocerciasis, it was noted that the current thresholds for stopping MDA and transitioning to a period of post-treatment surveillance (including by reaching the serological threshold of IgG4 antibody prevalence [against the Ov16 antigen] of <0.1% among children aged <10 years) are impractical for resource-limited settings and somewhat difficult to apply due to the low prevalence threshold required (at the upper 95% confidence limit), the large sample size necessary, and the variable and uncertain performance characteristics of available diagnostic tools [23]. Therefore, there is clear potential for modeling to support decisions around when to stop MDA.

There were similar discussions related to trachoma and the uncertainty around the active trachoma threshold for MDA: a trachomatous inflammation–follicular (TF) prevalence in children aged 1–9 years of greater than or less than 5% is used to inform the start or stop of MDA campaigns. The many challenges in interpreting survey results that are close to the 5% threshold were highlighted, knowing that there is “noise” around this threshold due to both sampling and diagnostic errors. One way in which modeling could help guide such decisions is to provide guidance on how the probability of being above/below the 5% threshold is influenced by survey results and differing levels of diagnostic sensitivity/specificity.

The discussions focused not only on start/stop decisions for MDA interventions but also those used for vector control. For VL, participants felt that modeling could help forecast what will happen if and when indoor residual spraying (IRS) campaigns are discontinued, thereby informing programmatic decisions. Modeling could also help guide decisions on how best to stop IRS, including number and size of sites, duration of follow-up, metrics to measure, and thresholds to start/stop/resume IRS efforts.

A final area identified for further studies related to start/stop decisions was the effort to incorporate additional geographical resolution to existing models. While this was a common theme across multiple diseases, it was illustrated by discussions around taeniasis/cysticercosis and the potential use of models to estimate the impact of spatially targeted versus population-wide control strategies. For example, programs would benefit from the ability to define target areas for focal *Taenia solium* control based on risk mapping, which would, in turn, help to direct resources, such as donated taenicides.

#### Never Treatment

A recurrent challenge across NTD interventions is the systematic lack of access to or acceptance of MDA among certain subpopulations, defined as “never treatment.” However, recent research has identified that it may be possible to measure the proportion of the population who have never been treated after a number of rounds of MDA [24–26]. Modeling efforts could focus on estimating the impact of a proportion of the population never receiving treatment on the achievement of the 2030 targets and elucidating how such impact may vary across different diseases and settings.

One example of where such models would be extremely valuable is the elimination of lymphatic filariasis (LF). While 17 of 72 LF-endemic countries have achieved EPHP [27], others continue to face important challenges, including due to gaps in treatment coverage and never treatment [28, 29]. Modeling that incorporates these factors has the potential to effectively support national and subnational programs in setting realistic coverage targets to achieve elimination and to evaluate the cost-effectiveness of any required additional interventions.

#### Cost

Decisions related to when to start and stop interventions, whom to treat and when, which population groups to sample and which tests to use, and how best to preserve public health gains, all have clear cost implications. The importance of such costs and other resources in influencing programmatic decisions was highlighted. To better support program planning and decision making, there is a clear need to further incorporate economic considerations into relevant models. For example, for STH, discussions revolved around the need to include cost estimates, including the cost of different interventions, and to evaluate the equity of programs. The likely utility of modeling STH scenarios across different time horizons and incorporating the cost of inappropriate treatment into the models to inform optimal intervention strategies was also highlighted. For taeniasis/cysticercosis, program managers pointed out the need for models to provide estimates of the effectiveness, cost-effectiveness, and time-to-attain “intensified control” targets in specific populations, which could be used to prioritize resources. For VL, there was a suggestion to use modeling to quantify the value of a “stratified” implementation and evaluation policy—for example, differentiating between low- and high-risk communities. In addition, it was suggested that modeling can be used to determine the appropriate incidence threshold for sustainable VL elimination. The discussions around both FBT and echinococcosis highlighted that modeling could be used to delineate appropriate, cost-effective packages of interventions for reducing infection intensity. For *gambiense* human African trypanosomiasis (gHAT), participants discussed how modeling has been used to analyze the cost-effectiveness of past interventions as well as recommend efficient future strategies [30]. It was raised that it can be challenging for non-modelers to quickly understand technical modeling outputs (eg, health economic results based on “willingness-to-pay” thresholds) and this is an important hurdle to overcome in communication if modeling is to be used to support decision making.

### Projecting the Impact of New or Adapted Interventions

Modeling can also be valuable to estimate the likely impact of new interventions across a range of NTDs. For onchocerciasis, pressing questions that were identified as likely to benefit from modeling included estimation of the impact of using novel medicines, particularly moxidectin, which was approved in 2018 by the US Food and Drug Administration for the treatment of individuals aged 12 years and older [31] (trials to determine dosing in children aged 4–11 years have also been recently completed [32]). Relevant information for guiding policy decisions includes not only the impact of moxidectin on onchocerciasis elimination timelines but also how benefits may differ depending on whether populations have previously received ivermectin MDA, or other interventions such as local vector control (although it should be noted that the use of local vector control is currently very limited in onchocerciasis programs). Additional insights into the optimal number of MDA rounds required to achieve elimination and the impact of alternative MDA strategies (eg, every 3 or 6 months, rather than annually) would likely also have important implications for decision making [33].

For LF, one of the recent tools available to accelerate achievement of the 2030 targets is the triple-drug combination of ivermectin, diethylcarbamazine citrate (DEC) and albendazole (collectively termed IDA) [34, 35]. However, the efficacy of IDA in phase IV trials has been variable and sometimes significantly lower than that observed in the initial clinical trials [36–41]. Modeling can help to evaluate how variable efficacy might affect the projected impact of IDA-based MDA programs or inform on the optimal number of MDA rounds in particular settings [42]. In populations in which MDA has had little impact on LF transmission, the additional intervention of adding DEC to salt (DEC-fortified salt) [43] is being considered. While DEC-fortified salt has been utilized successfully to control LF transmission in different regions for decades (including in China and India) [44, 45], practical considerations have impacted its use in other areas (such as Guyana and Haiti) [46, 47]. Nonetheless, modeling could help to evaluate the likely impact of this additional intervention in priority areas, with reasonable coverage assumptions for both MDA and DEC-fortified salt. Modeling could also be used to inform the implementation and/or development of alternative LF intervention strategies, including biannual albendazole MDA (the recommended strategy in areas of LF-loiasis co-endemicity) or the use of moxidectin (on its own or in combination with albendazole).

Beyond new and revised MDA regimens, modeling could also help guide other interventions. For trachoma, recent progress in *Chlamydia trachomatis* vaccine development [48] has raised the question of whether a trachoma vaccine could be technically feasible, and whether modeling could provide input on what characteristics such a vaccine would need in order to be cost-effective (eg, protection from infection, reduction in transmission, duration of effect, etc). There is also interest in trying to incorporate the contributions of facial cleanliness and environmental improvement (F&E) into existing trachoma models [49], although it was noted that there are challenges linked to the wide variation in F&E interventions and uncertainty around their impact on transmission [50].

Similarly, modeling could help estimate the impact of new reservoir- or vector-targeted interventions. As an example, the transmission cycle of VL in Brazil (which accounts for 97% of VL cases in the Americas) is very well established; dogs serve as the main reservoir host. Therefore, Brazil has started implementing insecticide-impregnated dog collars as an intervention, based on its success in other settings [51, 52]. Studies forecasting the impact of such strategies on vectors and on VL incidence, morbidity, and mortality, as well as quantifying cost-effectiveness, would be welcomed by NTD program managers. Finally, it was also noted that, due to the negative impacts of insecticide resistance on vector control strategies across a range of diseases (including VL, LF, and dengue), future studies incorporating insecticide-resistance modeling may help inform the design of more effective and sustainable disease-control programs.

### Incorporating Co-Endemicity

The potential use of moxidectin against both onchocerciasis and LF in co-endemic areas highlighted another main theme emerging from the meeting, which was the need for models that account for relevant co-endemicity and coinfections. For settings experiencing the dual burden of LF and onchocerciasis [53], questions were asked about whether modeling could help delineate the optimal strategies for MDA (including moxidectin) or how onchocerciasis interventions should proceed when LF MDA is stopped. It was also noted that ivermectin treatment can give rise to neurological serious adverse events (SAEs) in individuals with *Loa loa* infections [54]. Therefore, models that could help inform optimal test-and-not-treat strategies for MDA campaigns targeting LF and onchocerciasis in regions where loiasis is endemic would be invaluable [55].

Neurological SAEs are also a risk in individuals with concurrent neurocysticercosis who are treated with praziquantel as part of efforts to control and eliminate schistosomiasis [56]. Therefore, modeling to define co-endemic areas for these 2 diseases would support programmatic decisions on where to switch treatment to alternative antiparasitic medicines.

Coinfections with VL and human immunodeficiency virus (HIV) are a major concern in some countries [57], where the increased vulnerability of people with HIV to VL raises unique challenges for VL control and elimination efforts. There is a need for modelers and experts to incorporate coinfection into models that can then be used to better assess the likely impact of HIV coinfection and interventions on VL control and elimination.

### Monitoring, Evaluation, and Surveillance

Modeling can also help NTD programs to monitor progress, and optimize evaluation and surveillance strategies. One of the most pressing questions identified in multiple breakout sessions was how modeling could provide accurate insights and timely information on whether national programs are on track to achieve 2030 targets. Furthermore, participants highlighted the need for models to provide detailed insights into how good interventions need to be to reach set targets—for example, by helping to delineate required efficacy levels and effective coverage thresholds for the success of different interventions. One example discussed was Chagas disease, for which various studies have validated modeling approaches using routine seroprevalence surveys from some countries to assist the monitoring of 2030 goals in the region and estimate both acute and chronic disease burden at the subnational level [58–60]. Such approaches need to be expanded and validated for all Chagas-endemic countries. For VL, a range of important questions in these domains were identified, including about the benefits of generating improved estimates of the global, regional, and national burden of disease, in collaboration with the Global Burden of Disease Study [5]. Such estimates are important for decision making across a range of NTDs, including by providing baselines to facilitate evaluation of whether case detection thresholds are being met and assessing the likelihood for the occurrence of new cases or transmission in new areas.

Other heavily discussed topics were post-validation (for diseases that aim at EPHP) and post-verification (for those aiming at EOT) surveillance, and how modeling could assist with decisions about what to do when control/elimination targets are reached. For example, for LF, there is a need for modeling to support national programs in designing and evaluating post-validation surveillance, including consideration of the best ways to include newer methods such as molecular xeno-monitoring (capture and testing of disease vectors). Currently, different national programs are considering distinct mechanisms for maintaining surveillance in a cost-efficient way and it is important to evaluate the potential utility of these strategies and the opportunities for integrated surveillance across multiple diseases. Similarly, for onchocerciasis, the optimal duration and frequency of post-elimination surveillance remain unclear, and determining this could be the focus of future studies. For STH, the design of a practical framework for surveillance to detect resurgence would likely benefit from the inclusion of modeling insights. For yaws, which is targeted for eradication, there is also a need to design suitable surveys to identify whether previously endemic countries still carry disease burden. Certifying local yaws elimination is complicated by the cross-reactivity of yaws antigens with those of the causative agent of syphilis, and modeling may be used to estimate the expected serological age-prevalence profiles given the background signal from syphilis.

Other relevant questions identified across various diseases included how to define endemicity (such as in taeniasis) and how to define the appropriate methods and targets for monitoring and evaluation that would help to steer control programs (including for FBTs and echinococcosis).

## THE NEED FOR NATIONAL/SUBNATIONAL MODELS AND IN-COUNTRY CAPACITY

In addition to the priority questions defined above, participants reinforced the need for modeling to continue to move from global to regional, national, and subnational levels, to generate insights that can support programmatic decisions under real-world conditions. This call echoes the 2021–2030 road map and its calls to accelerate programmatic action and facilitate country ownership [2]. The importance of this progression to subnational emphasis was discussed across virtually all diseases but illustrated by the discussion on STH, where the need for global models to be turned into tactical, subnational models that include local data (such as treatment coverage and adherence) was stressed due to its importance for local decision making. Similarly, discussions on trachoma described how modeling the potential benefits of increased frequency of MDA in districts with persistent TF has been informative but noted that such studies are mainly based on generalized scenarios. Therefore, they could be improved by incorporating local context, which would facilitate interpretation of the results and their application to specific districts. For gHAT, which has an extremely low prevalence but is highly focal, participants agreed on the need to have even more granular spatial scales than health districts as highly geographically targeted strategies are operationally desirable. Broadly, it was agreed that there is a need for NTD models to account for within-country heterogeneity and provide insights into how to best intervene in places that present particular challenges to disease control and elimination. Another important related theme emerging from these discussions was the need to validate models prospectively, which will include the development of methods to quantify the reliability of existing models.

This transition to studies that can better inform local programs requires not only different models but also additional adaptations, including in-country capacity strengthening. For example, national onchocerciasis elimination committees should be better integrated into current frameworks, where they play important roles in providing input into models and access to local data, and help with model interpretation and use. The breakout session on trachoma also discussed potential paths towards implementation of more locally relevant models, including the development of new interfaces that can be customized with national data under a range of scenarios and/or the involvement of dedicated in-country modelers with appropriate training and coding knowledge. One possibility discussed was the engagement of WHO Collaborating Centers and academic groups within countries to increase capacities for modeling. There was also debate about the potential role of funders in financing positions that bridge the current gap between modelers and in-country programs. Such posts could also serve an important role in ensuring accountability and measuring progress, helping to assess how countries are taking ownership of different programs and whether they are on track to meet national targets.

## CONCLUSIONS

The meeting between WHO, the NTD Modelling Consortium, and other relevant stakeholders delineated multiple ways in which mathematical modeling has and can continue to inform programs focusing on the control, elimination, and eradication of NTDs. In order to achieve the ambitious targets set out in the 2021–2030 road map, future modeling studies should address the priority questions identified (summarized in Figure 1 and Table 2) and continue to move towards national and subnational models, which will support better programmatic action that incorporates local context [61]. Importantly, progress in these areas could be accelerated by expanding the ranks of modelers working on NTDs, particularly modelers based in NTD-endemic countries who are familiar with local transmission conditions and parameters, and who regularly interface with local public health decision-makers [62–64].

**Table 2.** Priority Neglected Tropical Diseases (NTD) Modeling Questions Identified by the World Health Organization, the NTD Modelling Consortium, and Other Stakeholders

**Modeling the impact of the COVID-19 pandemic**
Examples: What is the impact of reduced availability of medicines that resulted in missed rounds and/or reduced coverage of MDA campaigns? What are the best strategies to mitigate the impact of the above? Should planned impact surveys go ahead in light of variable coverage levels?
**Incorporating real-life challenges**
***Start and stop decisions***
Examples: What are appropriate levels of infection prevalence (including seroprevalence) for recommending the start of MDA campaigns? When, where, and how should treatment be expanded to nonpriority groups? What is the optimal number and frequency of MDA rounds, and how do these vary by transmission setting? What are appropriate levels of infection prevalence (including seroprevalence and infection prevalence in vectors) to stop interventions? What will happen if and when vector control campaigns are discontinued? What are the optimal ways to handle uncertainty (including in start/stop thresholds)?
***Never treatment***
Examples: What is the impact of a proportion of the population never receiving treatment on the achievement of the 2030 targets? How does the impact of never treatment vary across different diseases and settings?
***Cost***
Examples: How should costs of different interventions be included into models? How can models be used to estimate the cost-effectiveness of attaining “intensified control” targets in specific countries and regions? How can models be used to identify appropriate, cost-effective packages of interventions?
**Projecting the impact of new interventions**
Examples: What is the impact of novel drugs and/or vaccines on transmission and control, and how does it change across settings, including when used alone vs in combination with existing strategies? What is the impact of alternative treatment regimes, including changes to treatment frequency?
**Incorporating coinfections**
Examples: Can models be expanded to account for coinfections? How can models be used to help delineate optimal interventions in settings with multiple diseases? How can models help design and evaluate optimal test-and-treat and test-and-not-treat strategies?
**Monitoring, surveillance, and evaluation**
Examples: Are different NTD programs on track to achieve 2030 targets? Should interventions be intensified or additional measures be implemented to reach 2030 targets? What is needed to generate contemporary rigorous estimates of the global, regional, and national burden of disease for the various NTDs? Are burden estimates needed for surveillance and health planning? What is performance and cost-effectiveness of different post-validation or post-elimination surveillance strategies? How can modeling help to define operational “intensified control” targets? Can models help define the appropriate methods and targets for monitoring and evaluation? What is the potential value of integrated surveillance using methods such as molecular xeno-monitoring?

Abbreviations: COVID-19, coronavirus disease 2019; MDA, mass drug administration.

## Supplementary Data

Supplementary materials are available at *Clinical Infectious Diseases* online. Consisting of data provided by the authors to benefit the reader, the posted materials are not copyedited and are the sole responsibility of the authors, so questions or comments should be addressed to the corresponding author.

## Supplementary Material

ciae082_Supplementary_Data

## Notes

***Acknowledgments.*** The authors are grateful, as always, to the academic partners, field staff, and neglected tropical diseases (NTD)-affected populations who generate primary data and invaluable insights that are generously made available to facilitate modeling work. They also thank Beth Bruce and Leena Yousef for their support of the meeting.

***Financial support***. This work was supported by the Bill & Melinda Gates Foundation grant to the Neglected Tropical Diseases Modelling Consortium (INV-030046). E. H. C., C. N. D., C.-I. H., S. A. S., and K. S. R. acknowledge funding from the Bill & Melinda Gates Foundation to the HAT Modelling and Economic Predictions for Policy project (INV-005121). M.-G. B., K. K., M. D., and R. A. also acknowledge funding from the MRC Centre for Global Infectious Disease Analysis (MR/R015600/1), jointly funded by the UK Medical Research Council (MRC) and the UK Foreign, Commonwealth & Development Office (FCDO), under the MRC/FCDO Concordat agreement and also part of the EDCTP2 program supported by the European Union.

***Supplement sponsorship.*** This article appears as part of the supplement “New Tools and Nuanced Interventions to Accelerate Achievement of 2030 Roadmap for Neglected Tropical Diseases,” sponsored by funding of Professor T. Déirdre Hollingsworth's research by the Li Ka Shing Foundation at the Big Data Institute, Li Ka Shing Centre for Health Information and Discovery, University of Oxford; and funding of the NTD Modelling Consortium by the Bill & Melinda Gates Foundation (INV-030046).
