# Supplementary material for: Accelerating Progress Towards the 2030 Neglected Tropical Diseases Targets: How Can Quantitative Modeling Support Programmatic Decisions?
Source: Clin Infect Dis. 2024 Apr 25;78(Suppl 2):S83–92. doi: 10.1093/cid/ciae082 (PMC11045030; doi:10.1093/cid/ciae082)
Supplement: ciae082_Supplementary_Data [file ciae082_supplementary_data.docx]

**Supplementary materials**

**Supplementary materials 1. Meeting agenda**

**Day 1**

***First plenary (Chair: Jonathan King)***

| **Time** | **Topic** | **Speaker** |
| --- | --- | --- |
| 09:00-09:05 | Opening and meeting purpose | Chair |
| 09:05-09:28 | Introductions | All |
| 09:28-09:30 | Summary of declarations of interest | Chair |
| 09:30-09:45 | Progress towards the 2030 goals, challenges and opportunities for acceleration | Gautam Biswas |
| 09:45-09:50 | BMGF perspective on the importance of modelling to achieve the 2030 goals | Simon Brooker |
| 09:50-10:00 | Providing modelling to support decision making to accelerate progress on NTDs: | Déirdre Hollingsworth |
| 10:00-10:45 | Endorsement: what makes a model a WHO model? Should we establish a process? | Anthony Solomon and Andreia Vasconcelos |
| 10:45-11:00 | Aims of breakout groups | Anthony Solomon |

***Disease group 1 breakouts***

| **Session** | **Modellers** | **WHO staff** |
| --- | --- | --- |
| 1A. Rabies | Hampson, Tildesley | Abela-Ridder, Bote, Solomon |
| 1B. Leishmaniases (VL & CL) | Hollingsworth, Coffeng, Medley, Prada, de Vlas | Ruiz Postigo, Jain, Dagne, Elkhoury |
| 1C. STH and SCH | Anderson, Keeling, Stolk, Medley, Harrison, Fronterrè, Mbah, Kanyi, Schwehm, Toor, Philips, Tchuem, de Vlas, Chuenté, French, Kura, Coffeng, Spencer | Montresor, Mupfasoni, Garba, Mbabazi |

***Disease group 2 breakouts***

| **Session** | **Modellers** | **WHO staff** |
| --- | --- | --- |
| 2A. Lymphatic filariasis | Hollingsworth, Stolk, Anderson, Bilal, de Vlas | King |
| 2B. Onchocerciasis | Stolk, Walker, Michael, Basáñez, Lietman, James, Coffeng, de Vlas, Anderson | Rebollo, Mupfasoni |
| 2C. FBT, taeniasis, echinococcosis | Chitnis, Braae, Harrison, Prada, Basáñez, Dixon-Zegeye | Abela-Ridder, Bote |
| 2D. Chagas disease (virtual) | Cucunubá, Dobson, Cardozo, Basáñez, Ledien | Albajar Vinas |
| 2E. Yaws (virtual) | Dyson, Marks, Mitjà | Asiedu, Pathak, Dagne, Solomon |

**Day 2**

***Disease group 3 breakouts***

| **Session** | **Modellers** | **WHO staff** |
| --- | --- | --- |
| 3A. Leprosy (virtual) | Medley, Blok, Richardus, Davis, Raheem, Pannikar, Nobre, Hollingsworth | Asiedu, Pemmaraju, Dagne, Jain |
| 3B. Dengue | Chitnis, Pigott, Salje, Ferguson, Cummings, Rodriguez-Barraquer, Lessler, Brady | Velayudhan, Yadav, Zhong, Bezerra, Al-Eryani |
| 3C. Trachoma | Porco, Borlase, Blumberg, Prada, Giorgi, Sasanami, Lietman, Srivathsan | Solomon, Kello |
| 3D. Human African trypanosomiasis | Keeling, Rock, Castaño, Chitnis, Antillon, Hargrove, Crump, Dyson, Huang , Sutherland, Davis | Franco Minguell, Priotto |

***Second plenary (Chair: Andreia Vasconcelos)***

| **Time** | **Topic** | **Speaker** |
| --- | --- | --- |
| 13:00-13:30 | Expanding NTD modelling capacity in endemic countries | Thumbi Mwangi |
| 13:30-14:00 | Sub-national projections | Simon Spencer |
| 14:00-14:15 | Impact and recovery from COVID-19 | Déirdre Hollingsworth |
| 14:15-14:30 | Resolving onchocerciasis models differences | Basáñez & Stolk |
| 14:30-15:00 | Modelling long-term trends in morbidity | Jon Mosser |
| 15:15-15:45 | NTD endgame – future strategies | Aysu Uygur |
| 15:45-16:15 | Surveillance: optimal survey design for accelerating progress and detecting resurgence | Claudio Fronterrè |
| 16:15-16:45 | NTD impact module in the OneHealth Tool | Xiao Xian Huang |
| 16:45-17:30 | Cross-cutting themes, and next steps | Déirdre Hollingsworth, Anthony Solomon & all |

**Supplementary materials 2. Meeting participants**

In person and virtual. All attendees were invited to contribute to this publication.

**NTD Modellers**

Roy Anderson, Imperial College London, UK

Marina Antillon, Swiss Tropical and Public Health Institute, Switzerland

Maria-Gloria Basáñez, Imperial College London, UK

Shakir Bilal, College of Public Health, University of South Florida, USA

David Blok, Erasmus MC, The Netherlands

Seth Blumberg, University of California, San Francisco, USA

Anna Borlase, University of Oxford, UK

Oliver Brady, London School of Hygiene & Tropical Medicine, UK

Raiha Browning, University of Warwick, UK

Beth Bruce, University of Oxford, UK

Miriam Cardozo, CONICET and Universidad Nacional de Córdoba, Argentina

Soledad Castaño, Swiss Tropical and Public Health Institute, Switzerland

Nakul Chitnis, Swiss Tropical and Public Health Institute, Switzerland

Luc Coffeng, Erasmus MC, The Netherlands

Emily Crowley, University of Warwick, UK

Ron Crump, University of Warwick, UK

Zulma M. Cucunuba, Pontificia Universidad Javeriana, Colombia

Derek Cummings, University of Florida, USA

Emma Davis, University of Oxford, UK

Christopher Davis, University of Warwick, UK

Peter Diggle, Lancaster University, UK

Matthew Dixon-Zegeye, Imperial College London, UK

Andrew Dobson, Princeton University, USA

Louise Dyson, University of Warwick, UK

Mike French, RTI International, USA

Claudio Fronterre, Lancaster University, UK

Emanuele Giorgi, Lancaster University, UK

John Hargrove, University of Stellenbosch, South Africa

Deirdre Hollingsworth, University of Oxford, UK

Ching-I Huang, University of Warwick, UK

Ananthu James, Erasmus MC, The Netherlands

Ebrima Kanyi, University of The Gambia, The Gambia

Samson Kiware, Pan-African Mosquito Control Association (PAMCA), Kenya

Klodeta Kura, Imperial College London, UK

Julia Ledien, Imperial College London, UK

Tom Lietman, University of California, San Francisco, USA

Michael Marks, London School of Hygiene & Tropical Medicine, UK

Graham Medley, London School of Hygiene & Tropical Medicine, UK

Amanda Minter, University of Oxford, UK

Oriol Mitjà, Germans Trias I Pujol Research Institute, Germany

Jonathan Mosser, Institute for Health Metrics and Evaluation, USA

Thumbi Mwangi, Centre for Epidemiological Modelling and Analysis (CEMA), Kenya

Martial Ndeffo-Mbah, Texas A&M University, USA

Mutono Nyamai, Centre for Epidemiological Modelling and Analysis (CEMA), Kenya

Vijaykumar Pannikar, Lilavati Hospital, Mumbai, India

David Pigott, Institute for Health Metrics and Evaluation, USA

Travis Porco, University of California, San Francisco, USA

Benedict Quao, Ghana Ministry of Health, Ghana

Raheema Abdul Raheem, The Maldives National University, The Maldives

Kat Rock, University of Warwick, UK

Isabel Rodriguez-Barraquer, University of California, San Francisco, USA

Henrik Salje, University of Cambridge, UK

Misaki Sasanami, Lancaster University, UK

Markus Schwehm, ExploSYS GmbH, Germany

Milena Simic, Lepra, UK

Simon Spencer, University of Warwick, UK

Robert Stawski, University of Essex, UK

Wilma Stolk, Erasmus MC, The Netherlands

Samuel Sutherland, University of Warwick, UK

Ariktha Srivathsan, University of California, San Francisco, USA

Mike Tildesley, University of Warwick, UK

Louis-Albert Tchuem Tchuenté, University of Yaoundé, Cameroon

Andreia Vasconcelos, University of Oxford, UK

Sake de Vlas, Erasmus MC, The Netherlands

Martin Walker, Royal Veterinary College, UK

**WHO Secretariat HQ/NTD**

Bernadette Abela-Ridder

Kingsley Asiedu

Gautam Biswas, Director a.i.,

Daniel Argaw Dagne, Unit Lead, NTD

Camilla Ducker

Albis Gabrielli

Amadou Garba

Xiaoxian Huang

Saurabh Jain

Jonathan King

Pamela Mbabazi

José Ramon Franco Minguell

Antonio Montresor

Priya Pathak

José Ruiz Postigo

Gerardo Priotto

Dieudonné Sankara

Anthony Solomon

Afework Tekle

Raman Velayudhan

Pedro Alvajar Vinas

**WHO Secretariat Regional Offices**

Ana Nilce Elkhoury, AM/PAHO

Amir Kello, AFRO

Sunghye Kim, WPRO

Zaw Lin, SEARO

Ana Lucianez, AM/PAHO

Pauline Mwinzi, AFRO

Santiago Nicholls, AM/PAHO

Mona Osman, EMRO

Venkata Ranganadha Rao Pemmaraju, SEARO

Martha Idalí Saboyá-Díaz, AM/PAHO

Aya Yajima, SEARO

**Observers**

Simon Brooker, Bill & Melinda Gates Foundation, USA

Kayla Laserson, Bill & Melinda Gates Foundation, USA

Arnaud Le Menach, Clinton Health Access Initiative (CHAI), USA

Alison Ower, END FUND, USA

Deepa Pindolia, Clinton Health Access Initiative (CHAI), USA

Jordan Tappero, Bill & Melinda Gates Foundation, USA

Jaspreet Turner, Wellcome Trust, UK

Bhupendra Tripathi, Bill & Melinda Gates Foundation, USA

Aysu Uygur, Bill & Melinda Gates Foundation, USA

**Supplementary materials 3. Main questions identified from different breakout sessions.**

| **Session: 1A. Rabies** |
| --- |
| - No notes |
| **Session: 1B. Leishmaniases** |
| - When might a VL outbreak occur after COVID-19-related program interruptions? - What would be the value of a “stratified” implementation and evaluation of policy (e.g., differentiating between low and high-risk villages within blocks)? - What is the impact of PKDL and HIV-coinfection on feasibility of control/elimination? - What is the potential impact of an improved PKDL diagnostic? - What will happen if and when IRS is discontinued? - What is the burden of VL, globally, regionally, and nationally? - What is the role of housing conditions? - How much (or too little) would a future decline in case numbers have to be to be sufficient (or insufficient and therefore require action)? - What is the risk of re-establishment of transmission in areas that have achieved 3 years of control (or longer)? - What is the risk of establishment of transmission in new areas (e.g., predict suitability for transmission based on remote sensing data)? - Is the 1 per 10,000 target a blessing in disguise? Is low-level transmission better than complete cessation of transmission? - Is it possible to predict geographical expansion within countries? - Is it possible to predict the risk of presence of vector (including environmental variables and weather-related variables) and how this will impact transmission? - How to evaluate the implementation of impregnated dog collars as an intervention? - How to best model VL/HIV coinfection? |
| **Session: 1C. Schistosomiasis and soil-transmitted helminthiases (SCH and STH)** |
| - How can we design cost-effective and optimized surveys to help re-evaluate epidemiological situation, especially following multiple years of MDA? - Can we model tailored MDA programmes that can maintain reduction in morbidity? - Can modelling help design a practical framework for surveillance to detect recrudescence? - When and how to expand treatment to adult populations? - What is the optimal number and frequency of MDA rounds to achieve 2030 goals, and how do these vary by transmission setting? - How best to incorporate morbidity into models, allowing the trade-offs between morbidity control and elimination to be considered? - Can cost estimates be included into modelling, including the cost of different delivery systems? - What is the importance of accurately measuring coverage and compliance, as inputs into the models? - How can global model be turned in tactical, sub-national models which include local data and are used for decision making? - Is it possible to devise modelling across different time horizons and incorporating the cost of inappropriate treatment into the models? - How can we consider spatial dynamics of transmission, in addition to the empirical spatial distribution of infection? |
| **Session: 2A. Lymphatic filariasis** |
| - What are realistic coverage targets to achieve the 2030 goals using old and new tools? - Can modelling help to evaluate the cost-effectiveness of additional interventions? - How might the variable efficacy of IDA observed in field trials (compared to that observed in early clinical trials) affect its projected impact, or the number of rounds required to achieve the threshold for halting MDA? - Can modelling help to design/evaluate alternatives to the use of antigenaemia for evaluating whether to halt treatment after IDA? - What is the impact of a proportion of the population never receiving treatment on the achievement of the 2030 goals and how does this vary by setting? - What would be the impact of adding DEC salt to MDA in priority areas, with reasonable coverage assumptions for both MDA and DEC salt? - What would be the potential impact of additional interventions, including the use of moxidectin, moxidectin + albendazole or the impact of twice-yearly albendazole regimes? - What is the estimated timeline to reach elimination targets and ideal monitoring strategy for twice-yearly albendazole MDA with and without LLIN distribution? - Can modelling support national programmes in designing and evaluating post-validation surveillance strategies (including by evaluating the potential utility of the various mechanisms that are being considered for maintaining surveillance in a cost-efficient way)? - What is the potential value of xeno-monitoring (capture and testing of disease vectors)? How should data from such activities be better interpreted and understood? |
| **Session: 2B. Onchocerciasis** |
| - What is the number and optimal frequency of MDA rounds required to achieve elimination of transmission according to epidemiological setting? - How can modelling help Onchocerciasis Elimination Mapping to delineate and identify (endemically stable) hypoendemic communities in need of Start MDA? - When should we stop MDA based on (current or revised) serological and entomological thresholds with a minimal risk of resurgence? - Can modelling help determine optimal durations for post-treatment surveillance according to setting? - What is the impact of alternative MDA strategies (e.g., six- or three-monthly; moxidectin) on accelerating timelines to elimination of transmission? - What is the role of movement of people and vectors in maintaining or re-introducing transmission? - In places with LF and onchocerciasis co-infections, what is the optimal strategy for onchocerciasis once LF MDA has stopped? - What is the likely impact of moxidectin on elimination timelines, and does the benefit differ between ivermectin naïve versus non-naïve (with intervention history) settings? - Can modelling help design optimal test and (non-)treat strategies for settings with *Loa loa* co-endemicity? - What is the optimal duration of post-elimination surveillance, especially considering the long worm lifespan? |
| **Session: 2C. Food-borne trematodes (FBT), taeniasis, echinococcosis** |
| FBT   - What are appropriate levels of prevalence and intensity for recommending MDA? - What is the relationship between the history of infection and morbidity? - How do we define operational “intensified control” targets? - What is the role of reservoir hosts in maintaining and intensifying transmission? - What are appropriate packages of interventions for reducing intensity of infection (for the different food-borne trematodes)?   *Taenia solium taeniasis/cysticercosis*   - How do we define endemicity? - What are the appropriate targets for monitoring and evaluation? - How do we define operational “intensified control” targets? - What is the effectiveness and cost-effectiveness and timelines to attain “intensified control” targets in specific countries? - What is the impact of targeted (spatially-targeted) vs. population-wide strategies? - Where are the target areas for control (with taenicide donations) based on risk mapping? - What are the target areas for control based on co-endemic areas with schistosomiasis? - What are the target areas for control based on areas with risk of adverse SAEs in co-endemic areas?   Echinococcosis   - What are the appropriate targets for monitoring and evaluation? - How do we define operational “intensified control” targets? - What are appropriate packages of interventions for reducing intensity of infection? - What are the drivers of the relationships between the prevalence of infection in different hosts with each other? - What are the optimal diagnostic tools to use for surveillance programs? |
| **Session: 2D. Chagas disease** |
| - How can we scale from within-country heterogeneity modelling (e.g., Colombia), to across continent heterogeneity (e.g., South and Central America)? - How can modelling help leverage the information provided by serological surveys to improve burden of disease estimates? - How can we best incorporate multiple transmission paths (vectorial, congenital, transfusional/ transplantational, oral) into comprehensive modelling efforts for Chagas disease to reach 2030 goals? - How rapidly is Chagas spreading from endemic regions in South and Central America to urban areas in Western countries as asymptomatic hosts migrate in search of jobs? - How quickly does Chagas bounce back when control breaks down due to COVID-19 or other economic constraints (e.g., Venezuela)? |
| **Session: 2E. Yaws** |
| - How can modelling inform the design of suitable sampling surveys to identify whether previously-endemic countries still carry disease burden? - Can modelling be used to estimate the expected serological age-prevalence where syphilis is endemic, to assist with certification of local yaws elimination? |
| **Session: 3A. Leprosy** |
| - Can models incorporate the impact of COVID-19, including the substantial drop on the number of new cases, to provide more realistic estimations on the progress of control programmes towards 2019 targets? - How can modelling help to elucidate transmission pathways? For example, what is the role of zoonotic transmission? - Can models help to evaluate the impact of non-completion of multidrug therapy (MDT) on ongoing transmission? - How can modelling best inform the verification of interruption of transmission, including at the subnational level? - What is the impact of migration on disease dynamics and on the timeframes towards elimination? - How do we incorporate the impact of new leprosy cases on women, including by exploring gender biases around access to services and the disproportionate discrimination against women with disabilities? - Which enhanced methods (e.g., contact tracing, post-exposure prophylaxis, mass prophylaxis, new serology tests) can be used to accelerate elimination and what is their potential value and/or impact on elimination timelines? |
| **Session: 3B. Dengue** |
| - How do we combine multiple data types (entomological, epidemiological, laboratory, molecular, environmental) into integrated risk management systems to stratify areas by transmission and outbreak risk in real time? - How do we best roll out new interventions (where, when, how to combine with current interventions)? - Which combinations of interventions work best in Integrated vector management? And how can we coordinate control for dengue, Zika and chikungunya? - How can programmatic data be more widely combined with seroprevalence surveys to better estimate the true burden of disease? Particularly now multiple arbovirus co-circulate, complicating clinical and serological diagnosis. - Can models be used to map gaps in surveillance and access to appropriate treatment to reduce case fatality rates? - How can we use the growing amount of entomological information available to understand what mosquito population heterogeneities matter and can this inform Wolbachia roll out strategy and better predict risk of dengue in Africa? - Can WHO take the lead in developing a new global database of vector control, insecticide resistance and vaccine interventions at a pivotal time as many countries begin to adopt new tools? - Can predictive models inform vaccine trial site selection and risk of progression to severe disease? - How can we accelerate the uptake of models into ministry of health decision making through, for example, real-time dashboards? – what are the needs for modellers, what are the priority outputs for decision makers? - How much has COVID-19 impacted surveillance, diagnosis and vector control capacities for Aedes-borne diseases? Is this disruption linked to the large outbreaks in 2022/23? How can models use more contemporary data from 2020-2023, including a big boost to sequencing capacity, to better understand the impact of COVID-19? - How will countries respond to changes in dengue transmission caused by projected changes in climate, urbanisation and travel? |
| **Session: 3C. Trachoma** |
| - How can models better make use of local data to improve the forecasting of the potential benefits of increased frequency of MDA in districts with persistent infection? - What is the likely impact of COVID-19 on control, including missed rounds and reduced coverage? - Can modelling help with decisions such as whether a planned impact survey should go ahead, given that coverage has been lower than 80%? - How best to incorporate uncertainty around the 5% TF threshold? - Can modelling provide input on what characteristics a trachoma vaccine may need in order to have an impact (e.g., protection from infection, reduction in transmission, duration of effect, etc.)? |
| **Session: 3D. Human African trypanosomiasis (HAT)** |
| gHAT   - If humans are surrounded by animals such as pigs, which are the biggest suspects in potential animal contribution to gHAT transmission, is this protective or risky for humans? - Can we explain why there are regions without much vertical intervention (active screening or vector control) where cases have been found to be decreasing in passive detection? - What is the effectiveness, cost-effectiveness and timelines associated with different vector control strategies (sterile insect technique, tiny targets, etc.)? - What would be the impact of treatment of sero-suspects with acoziborole? What about mass treating whole villages with suspected on-going transmission? Or whole health areas? - As the focus has shifted from EPHP to elimination of transmission, how reliable are zero cases as a measure of this?   rHAT   - Would there be a way of predicting rHAT outbreaks which can often take the system by surprise and can cause significant mortality?   Both gHAT and rHAT   - Data can be incomplete, unreliability and limited; can modelling deal with that and are there ways to identify areas where there are limitations? |
